# Treatment Evolution after COPD Diagnosis in the UK Primary Care Setting

**DOI:** 10.1371/journal.pone.0105296

**Published:** 2014-09-02

**Authors:** Keele E. Wurst, Yogesh Suresh Punekar, Amit Shukla

**Affiliations:** 1 Worldwide Epidemiology, GlaxoSmithKline R&D, Research Triangle Park, North Carolina, United States of America; 2 Global Health Outcomes, GlaxoSmithKline R&D, Uxbridge, Middlesex, United Kingdom; 3 Worldwide Epidemiology, GlaxoSmithKline R&D, Upper Providence, Pennsylvania, United States of America; University of Tübingen, Germany

## Abstract

**Rationale:**

To assess the treatment progression during the 24 months following a formal diagnosis of chronic obstructive pulmonary disease (COPD) in the UK primary care setting.

**Methods:**

A retrospective cohort of newly diagnosed COPD patients was identified in the Clinical Practice Research Datalink (CPRD) from 1/1/2008 until 31/12/2009. Maintenance therapy prescribed within the first 3 months of diagnosis and in the subsequent 3-month intervals for 24 months were analyzed. Treatment classes included long-acting β_2_-agonists (LABAs), long-acting muscarinic antagonists (LAMAs), inhaled corticosteroids (ICSs), and respective combinations. At each 3-month interval, discontinuation, switching, addition, and stepping down patterns were analyzed cumulatively for the first 12 months and over the 24-month of follow-up.

**Results:**

A total of 3199 patients with at least one prescription of a maintenance therapy at baseline and during 4^th^-6^th^ month interval were included in the analysis. At diagnosis (0–3 months), the most frequently prescribed maintenance therapy was LABA+ICS (43%), followed by LAMA (24%) and LABA+LAMA+ICS (23%). Nearly half the patients (LABA-50%, LAMA-43%) starting on a monobronchodilator had additions to their treatment in 24 months. Compared to other medications, patients starting on a LAMA were most likely to escalate to triple therapy in 24 months. Nearly one-fourth of the patients prescribed triple therapy at baseline stepped down to LABA+ICS (25%) or LAMA (31%) within 24 months.

**Conclusion:**

Disease progression is evident over the 24 months after COPD diagnosis, as more patients were prescribed additional maintenance therapy in the 24-month period compared to baseline. The changes in therapy suggest that it is difficult to achieve a consistently improved COPD disease state.

## Introduction

Chronic obstructive pulmonary disease (COPD) is “a preventable and treatable disease characterized by airflow limitation that is not fully reversible” [1]. It remains a major healthcare problem, and various guidelines have been created to aid in effective management of this disease [1]–[4]. International guidelines such as the Global Initiative for Chronic Obstructive Lung Disease (GOLD) strategy and local guidelines such as the National Institute for Health and Care Excellence (NICE) provide guidance to physicians in treating COPD [1].

Once COPD is diagnosed and its severity is established, pharmacological treatment aims to reduce symptoms and exacerbations [2], [3]. Short-acting β_2_-agonists (SABAs) are used on an as-needed basis for symptomatic relief in early stages. As COPD is a progressive condition, daily maintenance drugs are usually needed. GOLD and NICE guidelines suggest that bronchodilators such as β_2_-agonists and antimuscarinics form the mainstay of therapy [1], [2]. Changes in forced expiratory volume in 1 second (FEV_1_) following bronchodilator therapy can be small; however, the accompanying larger change in lung volume contributes toward reduction in perceived breathlessness [5]. Long-acting β_2_-agonists (LABAs) or long-acting muscarinic antagonists (LAMAs) are also used to reduce the number and severity of exacerbations [6]. Combination therapy produces a greater change in spirometry and symptoms in comparison with monotherapy [7]. A combination of LABA and LAMA is prescribed if symptoms persist even after individual bronchodilator monotherapy. Inhaled corticosteroids (ICSs) are added as anti-inflammatory agents to reduce the rate of exacerbation in patients with severe disease [2], [8], [9]. NICE guidelines in the UK indicate a stepwise progression from bronchodilator monotherapy to a combination therapy based on the level of post-bronchodilator FEV_1_ percent predicted. For patients with FEV_1_ ≥ 50%, a LAMA or a LABA is recommended as the initial choice. If the FEV_1_ is <50%, the treatment choice is between a LAMA or LABA+ICS. Patients starting on LAMA or LABA+ICS would then progress to triple therapy (LABA+LAMA+ICS) if exacerbations or breathlessness persist [2].

Numerous studies report less than optimal compliance with guidelines in patients with COPD [10]–[12]. Therefore, a primary care-based study investigating treatment evolution in the 24 months after COPD diagnosis may help to identify patterns in progression of therapy, which can be used to create targeted interventions and educational programs. The objective of this study was to assess the evolving treatment paradigm over 24 months following the first COPD diagnosis, including discontinuation, addition, and switching of therapy.

## Methods

### Study design

In this retrospective cohort study, we evaluated treatment patterns of patients with COPD identified from the primary care setting using the Clinical Practice Research Datalink (CPRD), which is the National Health Service (NHS) observational data and interventional research service [13]. The CPRD is jointly funded by the NHS, the National Institute for Health Research (NIHR), and the Medicines and Healthcare Products Regulatory Agency (MHRA) [13]. This study used the primary care electronic medical records database part of the CPRD, formerly known as General Practice Research Database (GPRD). The study analyzed anonymized electronic records, and the protocol WEUSKOP5904 was approved by the CPRD Independent Scientific Advisory Committee (ISAC).

Patients aged ≥40 years with the first diagnosis of COPD and at least 12 months of history preceding the diagnosis were included. The diagnosis was confirmed by spirometry (FEV_1_/FVC < 0.7; FVC, forced vital capacity) at COPD index date/cohort entry date (between January 1, 2008 until December 31, 2009). Patients were labeled as cases of newly diagnosed COPD only if they had no prior diagnosis of COPD ever in their records. Further, these patients were required to have at least a 24-month follow-up history, unless death occurred during the follow-up period. Subjects with an occurrence of an event code of a medical condition incompatible with COPD diagnosis at any time in their history were excluded. These events included lung or bronchial developmental anomalies, degenerative processes (cystic fibrosis, pulmonary fibrosis), bronchiectasis, pulmonary resection, or other significant respiratory disorders that can interfere with clinical COPD diagnosis or substantially change the natural history of the disease.

Patient characteristics such as age, gender, body mass index (BMI), smoking status, available Medical Research Council (MRC) dyspnea scale score, and prior diagnosed and/or treated comorbid conditions (according to the Charlson index) [14] any time in the patient record were assessed at and prior to the cohort entry date. COPD prescriptions were evaluated at cohort entry date and every 3 months thereafter for a total follow-up duration of 24 months. The 3-month intervals included were 0–3 (baseline), 4–6, 7–9, 10–12, 13–15, 16–18, 19–21, and 22–24 following the cohort entry date. Treatment classes included LABAs and LAMAs alone, in combination (LABA+LAMA), and with an ICS (LABA+ICS, LAMA+ICS, and LABA+LAMA+ICS). All multiple therapies, either in combination or as separate inhalers, were required to be prescribed on the same day or within a 30-day period. Each medication category was mutually exclusive, irrespective of the use of short-acting bronchodilators (SABDs). The first maintenance prescription for COPD (either single or combination within 30 days) in each 3-month period was recorded as the therapy for that period.

In order to assess the progression through individual maintenance therapy, patients prescribed one of the following six index therapies at baseline and at each 3-month interval were included: LABA, LAMA, LABA+LAMA, LABA+ICS, LAMA+ICS, and LABA+LAMA+ICS. ICS as a standalone treatment was excluded, as ICS is not licensed as monotherapy for COPD. Patients were categorized into a baseline treatment class based on their first prescription in the 0- to 3-month interval. Patients were considered to continue on this index therapy if they were prescribed the same therapy in subsequent 3 monthly time intervals. Patterns of first change in treatment (addition, discontinuation, step down, and switch from index therapy) were analyzed at each time interval and cumulatively in the first (1–12 months) and second year (0–24 months). Patients with any change in therapy were excluded in subsequent intervals, thereby restricting the analysis to patients continuing on index therapy, while recording their first change.

In addition, the time to first prescription of triple therapy by index therapy among all newly diagnosed patients was evaluated using a Kaplan-Meier curve. Exploratory analysis was performed using Cox proportional hazards models by excluding asthma and adjusting for factors associated with progression to triple therapy (airflow limitation, underweight BMI, and suspected exacerbations).

## Results

### Patient disposition and demographic characteristics

Among the cohort of 7881 newly diagnosed patients with COPD, 3925 (50%) were prescribed maintenance therapy during the baseline period (0–3 months after diagnosis). Of the 3925 patients, 80% (n = 3199) were prescribed a maintenance therapy at baseline and during the 4–6 month interval and were included in this analysis. These patients had a mean (SD) age of 67 (11) years. The cohort consisted of 47% women, 15% former smokers, and 36% current smokers. Additionally, 97% and 66% of patients had an available stage of lung function and MRC dyspnea scale score, respectively (Table 1). The demographic distribution did not differ substantially from the overall newly diagnosed cohort apart from the stages of lung function and MRC score, where a higher proportion of patients prescribed maintenance therapy (n = 3199) had percent-predicted FEV_1_ < 50% (35% vs 27%) and MRC score ≥ 3 (26% vs 21%).

**Table 1:** Patient disposition and demographic characteristics.

Patient Variable, n (%)	Total COPD Cohort
All COPD	3199 (100.0)
Age, mean (SD)	67.29 (10.7)
Female	1489 (46.55)
Smoking status - Current smoker	1155 (36.11)
Smoking status - Former smoker	480 (15.00)
BMI, kg/m^2^	27.05 (6.11)
Charlson Score Prior	532 (16.63)
Acute myocardial infarction	271 (8.47)
Congestive heart disease	203 (6.35)
Cerebrovascular disease	211 (6.60)
Depression	416 (13.00)
Anxiety	492 (15.38)
Asthma	1417 (44.30)
Cancer	392 (12.25)
Diabetes	447 (13.97)
Diabetes with complications	93 (2.91)
Severity COPD GOLD 1	448 (14.00)
Severity COPD GOLD 2	1557 (48.67)
Severity COPD GOLD 3	925 (28.92)
Severity COPD GOLD 4	185 (5.78)
MRC 1	394 (12.32)
MRC 2	870 (27.20)
MRC 3	558 (17.44)
MRC 4	237 (7.41)
MRC 5	42 (1.31)

Abbreviations: BMI, body mass index; COPD, chronic obstructive pulmonary disease; GOLD, Global Initiative for Chronic Obstructive Lung Disease stage of airflow limitation; MRC, Medical Research Council dyspnea scale; SD, standard deviation.

### Treatments at baseline

**Figure 1:**
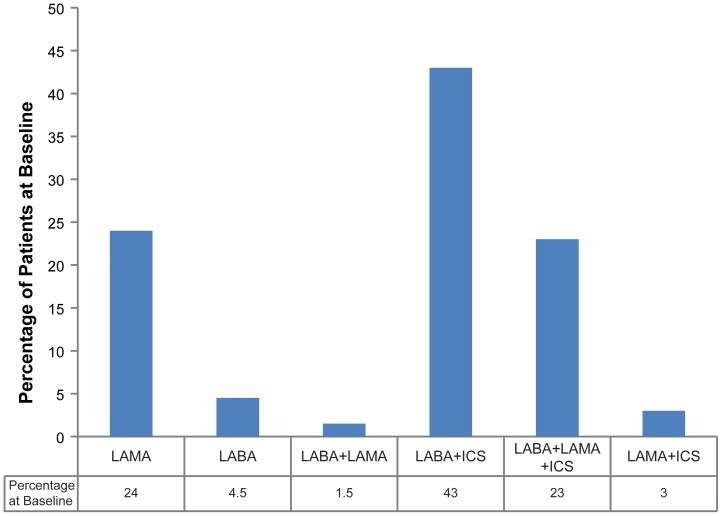
Maintenance prescriptions at diagnosis. Patients (n = 3199) prescribed maintenance therapy at baseline and 3–6 months excluding patients with no treatment, SABD alone, ICS, and others. Abbreviations: ICS, inhaled corticosteroid; LABA, long-acting β_2_-agonist; LAMA, long-acting muscarinic antagonist.

The frequency distribution of each type of prescribed therapy following a new diagnosis of COPD is shown in Figure 1. At diagnosis (0–3 months), the most frequently prescribed maintenance therapy was LABA+ICS (43%), followed by LAMA (24%) and LABA+LAMA+ICS (23%). The distribution of maintenance therapy was similar between patients prescribed a maintenance therapy at baseline (0–3 months, n = 3925) and those included in this analysis (n = 3199); data not shown.

### Changes in therapy over 24 months

#### Continuing on the same medication

**Figure 2:**
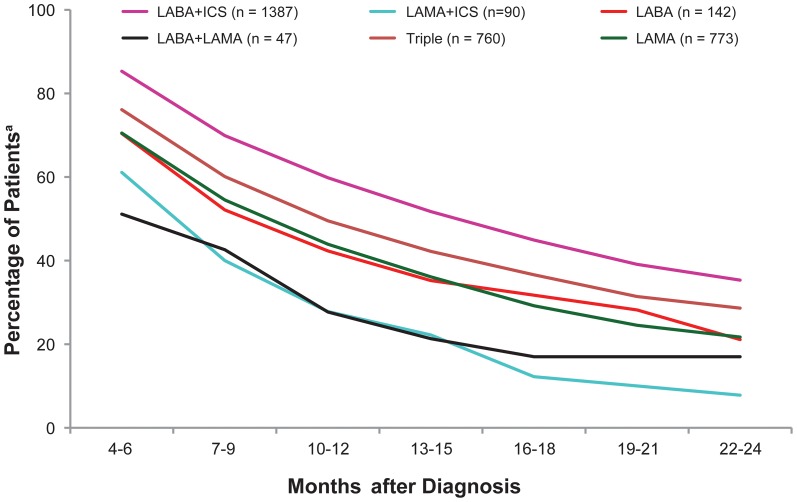
Proportions of patients continuing on the same treatment class for 24 months. ^a^ To be included in analysis, patients had to receive a prescription at each 3-month interval. Triple, LABA+LAMA+ICS. Abbreviations: ICS, inhaled corticosteroid; LABA, long-acting β_2_-agonist; LAMA, long-acting muscarinic antagonist.

Approximately one-quarter of the cohort was not prescribed their initial therapy again in the first 4–6 months following diagnosis; half of the cohort was not prescribed their initial therapy again after 12 months; and approximately three-quarters of the cohort was not prescribed their initial therapy again after 2 years (Figure 2).

**Figure 3:**
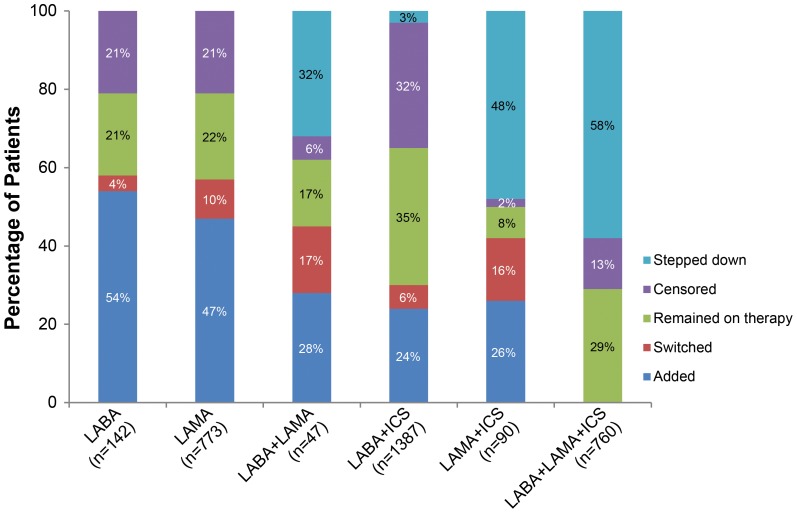
Percentage of changes in maintenance medications at the end of the 24 months of follow-up. Censored indicates no prescription for a COPD maintenance medication during a 3 month interval.

Patients on LABA+ICS combination demonstrated highest continuation, with 60% at interval 4 (10–12 months), and 35% at interval 8 (22–24 months) continuing on their index therapy. Patients on LABA and LAMA monotherapies had comparable continuation with 42% and 44% patients on the index treatment at interval 4 and 21% and 22% on index treatment at interval 8, respectively. The proportion of patients remaining on their initial therapy at the end of 24 months varied between initial medications prescribed. The proportions ranged from 8% for LAMA+ICS to 35% for LABA+ICS and about 25% for most other therapies (Figure 3). There were also a number of patients within each therapy group who were not prescribed a COPD maintenance therapy in the 3-month period following their initial prescription or first change in therapy (Figure 3).

#### Additions

Table 2 shows that a majority of additions occurred within the first 12 months of diagnosis. Among patients starting on a LABA or a LAMA therapy respectively, ICS (23% at 12 months, 28% at 24 months) and ICS+LABA (27% at 12 months and 33% at 24 months) were the most common additions within 12 and 24 months of follow-up. Other major additions within first 12 months included addition of ICS to LABA+LAMA starters (21% at 12 months, 28% at 24 months) and addition of a LAMA to ICS+LABA starters (17% at 12 months and 24% at 24 months) (Table 2).

**Table 2:** Proportions of patients adding to medication.

Treatment Class		N^a^	LABA	LAMA	LABA+ LAMA	LABA + ICS	LAMA + ICS	LABA+ LAMA+ ICS
LABA	0–12 months	142	-		8.5%	23.2%		5.6%
	0–24 months	142	-		14.8%	28.9%		6.3%
LAMA	0–12 months	773		-	3.0%		3.9%	26.8%
	0–24 months	773		-	4.5%		5.2%	33.2%
LABA+LAMA	0–12 months	47			-			21.3%
	0–24 months	47			-			27.7%
LABA+ICS	0–12 months	1387				-		16.9%
	0–24 months	1387				-		24.4%
LAMA+ICS	0–12 months	90					-	10.0%
	0–24 months	90					-	13.3%

a Number of patients starting/remaining on treatment at the beginning of the year.

Abbreviations: ICS, inhaled corticosteroid; LABA, long-acting β2-agonist; LAMA, long-acting muscarinic antagonist.

#### Switching or stepping down therapy

Few patients were prescribed another class of maintenance therapy within 24 months of diagnosis. Similar to additions, a majority of switches occurred within the 12 months of diagnosis (Table 3). A small percentage of patients switched from LABA to LAMA and from LAMA to LABA (Table 3).

**Table 3:** Proportions of patients switching to another medication or stepping down treatment.

Treatment Class		N^a^	LABA	LAMA	LABA+ LAMA	LABA + ICS	LAMA + ICS	LABA+ LAMA+ ICS
LABA	0–12 months	142	-	4.2%	NA	NA	0.0%	NA
	0–24 months	142	-	4.2%	NA	NA	0.0%	NA
LAMA	0–12 months	773	0.0%	-	NA	7.6%	NA	NA
	0–24 months	773	0.0%	-	NA	9.4%	NA	NA
LABA+LAMA	0–12 months	47	12.8%	17.0%	-	12.8%	4.3%	NA
	0–24 months	47	12.8%	19.1%	-	12.8%	4.3%	NA
LABA+ICS	0–12 months	1387	2.2%	3.7%	0.0%	-	0.0%	NA
	0–24 months	1387	2.6%	4.8%	0.0%	-	0.0%	NA
LAMA+ICS	0–12 months	90	0.0%	44.4%	0.0%	6.7%	-	NA
	0–24 months	90	0.0%	47.8%	0.0%	7.8%	-	NA
LABA+LAMA+ ICS	0–12 months	760	0.3%	23.0%	0.4%	19.2%	0.9%	-
	0–24 months	760	0.3%	31.3%	0.7%	24.5%	1.3%	-

a To be included in analysis, patients had to receive a prescription at each 3-month interval.

Abbreviations: ICS, inhaled corticosteroid; LABA, long-acting β2-agonist; LAMA, long-acting muscarinic antagonist.

Patients prescribed more than one maintenance medication in combination were included in the stepped down therapy analysis. Among patients starting on dual therapies, majority of step downs occurred in patients starting on LAMA+ICS therapy with 44% stepping down to LAMA monotherapy within first 12 months. Several patients starting on LABA+LAMA combination also stepped down to a LABA (13%) or a LAMA (17%) monotherapy within 12 months. Majority of patients prescribed triple therapy at baseline stepped down to LABA+ICS (19%) or LAMA (23%) during the 24-month follow-up (Table 3).

#### Progression to triple therapy

Results from the Kaplan-Meier survival analysis showed that the proportion of patients that progressed to triple therapy varied depending on the initial medication prescribed (Table 4). At 1 year, only 7% of patients who had not been prescribed COPD therapy initially had progressed to triple therapy. Among those prescribed LABD or an ICS combination initially, 32% and 25% of the patients, respectively, had progressed to triple therapy at 1 year, and 46% and 39% of the patients, respectively, had progressed to triple therapy at 2 years. When asthma was excluded and factors associated with progression to triple therapy (airflow limitation, underweight BMI, and suspected exacerbations) were adjusted for, the same trend was observed, ie, patients who received LABD or an ICS combination initially were prescribed triple therapy at a faster rate than those with other initial therapies (data not shown).

**Table 4:** Percent of patients that progressed to triple therapy within 2 years by index treatment prescribed.

Initial prescription at new COPD diagnosis, n	Progressed to triple therapy at 365 days, n (%)	Progressed to triple therapy at 730 days, n (%)
SABD, n = 1804	174 (10)	353 (20)
LABD (LAMA or LABA), n = 1232	397 (32)	568 (46)
ICS, n = 724	78 (11)	132 (18)
ICS combination (LABA+ICS or LAMA+ICS), n = 1856	461 (25)	723 (39)
No treatment, n = 1181	88 (7)	182 (15)

Abbreviations: COPD, chronic obstructive pulmonary disease; ICS, inhaled corticosteroid; LABA, long-acting β_2_-agonist; LABD, long-acting bronchodilator; LAMA, long-acting muscarinic antagonist; SABD, short-acting bronchodilator.

## Discussion

In this study, we evaluated the treatment paradigm evolving over 24 months after the first diagnosis of COPD in the UK general practice. Maximum variability in treatment was observed within the first 6 months, with approximately 15% to 49% of patients changing their initial therapy. Beyond this period, patients gradually stabilized on their respective treatments, with limited changes over the next 18 months of the follow-up. Majority of changes beyond 6 months included addition of treatments with limited switches. In addition, the index treatment was discontinued for most patients within the 24 months of diagnosis irrespective of the treatment class. This gradual yet significant change in therapy may indicate the difficulty in achieving a stable disease state in a progressive disease such as COPD. Thus, this observed change from initial therapy, with addition or switching, may suggest suboptimal disease control.

The results of this study are consistent with other studies that examined treatment patterns in COPD. Overall retention of treatment was low across all medications, although differences were observed among different treatment classes. In a study using the Ontario Drug Benefit Program database, Cramer et al demonstrated that 15–63% of patients continued on their index drug for more than six months [15]. Corresponding estimate in our study was 40–70%. Our results were also consistent with Cramer et al at 12 and 18 months [15].

The Cramer et al study found that patients stayed on tiotropium treatment significantly longer than other daily medications. In contrast, the treatment class with maximum retention in our study was LABA+ICS. The patients in the current study could represent COPD patients with concomitant yet undiagnosed asthma as 44% of the patients prescribed LABA+ICS in the total cohort had at least one asthma diagnosis in their record. These patients may also have had exacerbations in their previous history and received benefit from the ICS.

Guidelines suggest adding an ICS as anti-inflammatory agents to reduce the rate of exacerbation in patients with severe disease [2], [8], [9]. Our analysis showed that within 24 months, ICS as a single inhaler or LABA+ICS combination inhaler, was added for 35% and 38% of patients starting on a LABA and a LAMA monotherapy, respectively. This indicates that a substantial number of patients on bronchodilator monotherapy may require an ICS to possibly manage their risk of exacerbations. Similarly, a substantial number of patients starting on a LABA or a LAMA also added a second bronchodilator (with or without an ICS) within 24 months of COPD diagnosis. This may also suggest the progressive nature of COPD requiring additional pharmacotherapy to maintain lung capacity, though the data only allow generation of hypotheses due to lack of physician recording of the rationale behind medication changes.

Many patients starting on a long-acting bronchodilator (LAMA or LABA) or an ICS combination that includes a long-acting bronchodilator received a prescription for triple therapy by the end of the 2-year study period. This finding was observed with two different analyses: when patients were analyzed from their first maintenance therapy until first change in prescription, and when patients were followed up throughout the study period. Thus, the results suggest that COPD treatment evolution is progressive and patients probably need more than one maintenance medication to achieve symptom relief and/or prevent exacerbations.

Stepping down therapy may be reflective of prescriptions of multiple inhalers used intermittently. Among triple therapy initiators who stepped down during the follow-up period, majority of patients stepped down to a LAMA or an LABA+ICS combination. This study only addressed first change in therapy and perhaps these patients may have been prescribed triple therapy again at a later date.

Study limitations included the fact that GPRD only captures diseases diagnosed in the primary care setting. Also, there may be a possibility of misclassification of drug exposure because the GPRD provides information on prescribed rather than dispensed medications. However, audits of electronic medical records in the UK have shown a relatively high concordance of dispensing to prescription (99.7% of prescriptions tracked were recorded by the electronic patient record during a month) [16]. Only patients alive for 24 months were included in the analysis thus the findings may not be representative of most severe COPD patients.

## Conclusion

Challenges in optimally managing the condition were evident over the 24 months after COPD diagnosis, as more patients compared to baseline were prescribed additional maintenance therapy in the 24-month period. A high proportion of patients were switched or discontinued from their index treatment within 24 the months of diagnosis. Patients starting on LAMA or LABA+ICS therapy were most likely to change to triple therapy. Thus, the results suggest that COPD treatment evolution is progressive and patients likely need more than one maintenance medication to provide symptom relief and/or prevent exacerbations. The changes in therapy observed in this real world cohort suggest that it is difficult to achieve a consistently improved COPD disease state over two years and the unmet need remains.

## References

[1] GOLD strategy for the diagnosis, management, and prevention of chronic obstructive pulmonary disease. Avaliable: http://www.goldcopd.org/uploads/users/files/GOLD_Report2014_Feb07.pdf. Accessed 2014 Jun 1.

[2] National Institute for Health and Clinical Excellence.NICE clinical guideline 101. Available: http://www.nice.org.uk/nicemedia/live/13029/49397/49397.pdf. Accessed 2013 Jul 26.

[3] Rabe KF, Hurd S, Anzueto A, Barnes PJ, Buist SA, et al (2007) Global strategy for the diagnosis, management, and prevention of chronic obstructive pulmonary disease: GOLD executive summary. Am J Respir Crit Care Med 176: 532–555. 17507545 10.1164/rccm.200703-456SO

[4] Bellamy D, Bouchard J, Henrichsen S, Johansson G, Langhammer A, et al (2006) International Primary Care Respiratory Group (IPCRG) Guidelines: management of chronic obstructive pulmonary disease (COPD). Prim Care Respir J 15: 48–57. 16701758 10.1016/j.pcrj.2005.11.003 PMC6730681

[5] Martin J, Carrizo S, Gascon M, Sanchez A, Gallego B, et al (2001) Inspiratory Capacity, Dynamic Hyperinflation, Breathlessness, and Exercise Performance during the 6-Minute-Walk Test in Chronic Obstructive Pulmonary Disease. Am J Respir Crit Care Med 163: 1395–1399. 11371407 10.1164/ajrccm.163.6.2003172

[6] Wedzicha J, Decramer M, Seemungal T (2012) The role of bronchodilator treatment in the prevention of exacerbations of COPD. Eur Respir J 40: 1545–1554. 22835613 10.1183/09031936.00048912 PMC3511775

[7] van der Molen T, Cazzola M (2012) Beyond lung function in COPD management: effectiveness of LABA/LAMA combination therapy on patient-centred outcomes. Prim Care Respir J 21: 101–108. 22222945 10.4104/pcrj.2011.00102 PMC6547888

[8] American Thoracic Society/European Respiratory Society. Standards for diagnosis and management of patients with COPD. Available: http://www.thoracic.org/clinical/copd-guidelines/resources/copddoc.pdf. Accessed 2013 Nov 22.

[9] Osthoff M, Jenkins C, Leuppi JD (2013) Chronic obstructive pulmonary disease - a treatable disease. Swiss Med Wkly 143: 0. 10.4414/smw.2013.13777 23592218

[10] Salinas GD, Williamson JC, Kalhan R, Thomashow B, Scheckermann JL, et al (2011) Barriers to adherence to chronic obstructive pulmonary disease guidelines by primary care physicians. Int J Chron Obstruct Pulmon Dis 6: 171–179. 21468169 10.2147/COPD.S16396 PMC3064423

[11] Jochmann A, Neubauer F, Miedinger D, Schafroth S, Tamm M, et al.. (2010) General practitioner's adherence to the COPD GOLD guidelines: baseline data of the Swiss COPD Cohort Study. Swiss Med Wkly; April 21. 10.4414/smw.2010.13053 20407960

[12] Perez X, Wisnivesky JP, Lurslurchachai L, Kleinman LC, Kronish IM (2012) Barriers to adherence to COPD guidelines among primary care providers. Respir Med 106: 374–381. 22000501 10.1016/j.rmed.2011.09.010 PMC3377746

[13] Clinical Practice Research Datalink. Available: http://www.cprd.com/home/. Accessed 2012 Oct 15.

[14] Khan NF, Perera R, Harper S, Rose PW (2010) Adaptation and validation of the Charlson Index for Read/OXMIS coded databases. BMC Fam Pract 11: 1. 20051110 10.1186/1471-2296-11-1 PMC2820468

[15] Cramer JA, Bradley-Kennedy C, Scalera A (2007) Treatment persistence and compliance with medications for chronic obstructive pulmonary disease. Can Respir J 14: 25–29. 17315055 10.1155/2007/161652 PMC2690446

[16] Hassey A, Gerrett D, Wilson A (2001) A survey of validity and utility of electronic patient records in a general practice. BMJ 322: 1401–1405. 11397747 10.1136/bmj.322.7299.1401 PMC32256

